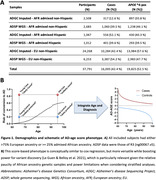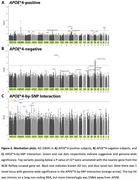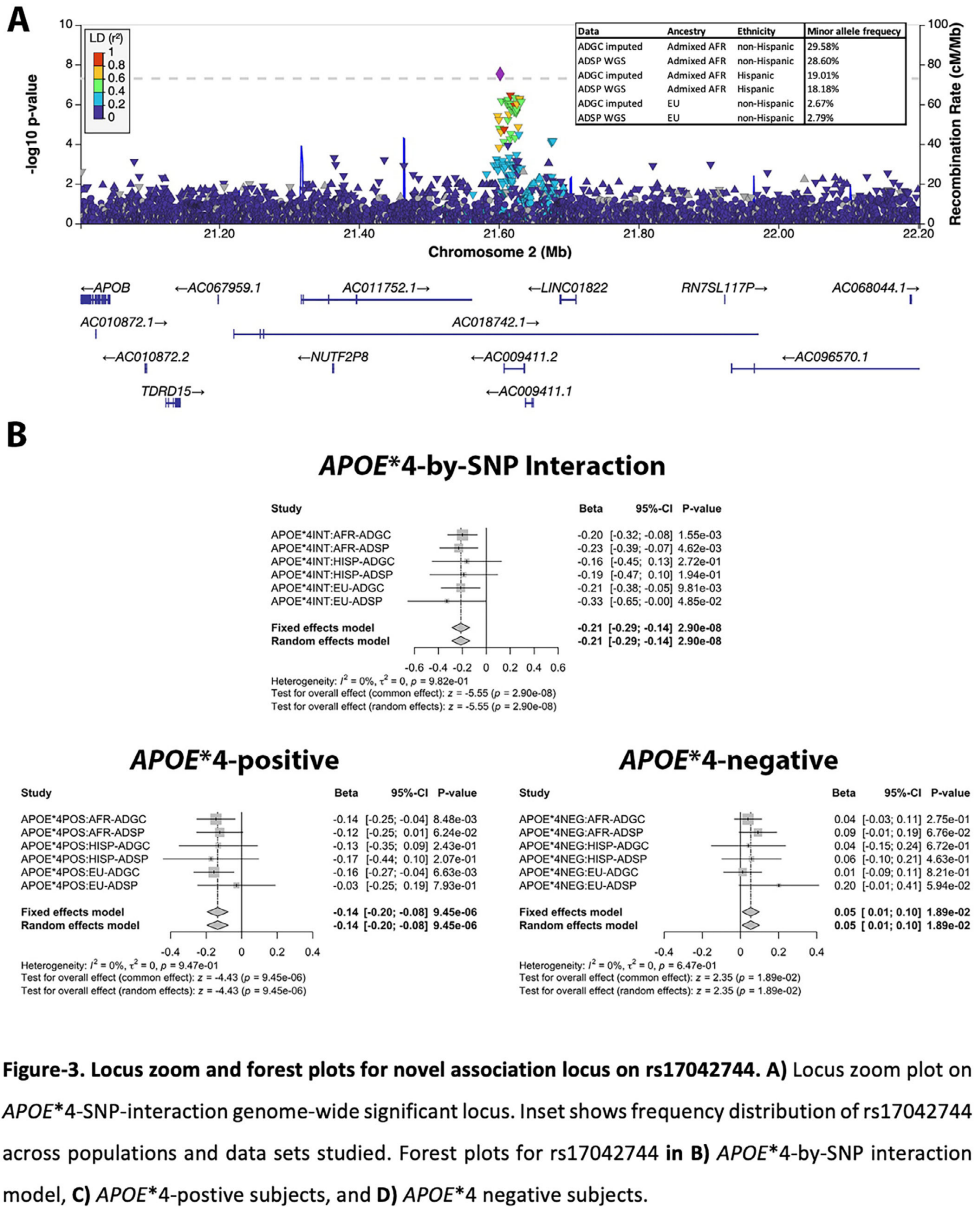# Trans‐ancestry GWAS for Alzheimer’s Disease Reveals a Novel Locus interacting with APOE*4

**DOI:** 10.1002/alz.090370

**Published:** 2025-01-03

**Authors:** Michael E. Belloy, Danielle M. Reid, Yann Le Guen, Valerio Napolioni, Michael D Greicius

**Affiliations:** ^1^ Washington University in Saint Louis, Saint Louis, MO USA; ^2^ Stanford University, School of Medicine, Stanford, CA USA; ^3^ Stanford University, Stanford, CA USA

## Abstract

**Background:**

APOE*4 is the strongest genetic risk for late‐onset Alzheimer’s disease (AD), but other genetic loci may counter its detrimental effect, providing therapeutic avenues. Expanding beyond non‐Hispanic White subjects, we sought to additionally leverage genetic data from non‐Hispanic and Hispanic subjects of admixed African ancestry to perform trans‐ancestry APOE*4‐stratified GWAS, anticipating that allele frequency differences across populations would boost power for gene discovery.

**Method:**

Participants were ages 60+, of European (EU; ≥75%) or admixed African (AFR; ≥25%) ancestry, and diagnosed as cases or controls. Genetic data were available from SNP arrays imputed to TOPMed or whole genome sequencing (WGS). Genome‐wide association studies (GWAS) were performed per data type, APOE*4 status, and Hispanic/non‐Hispanic status (**Figure‐1A**), requiring genotyping rate ≥80% and minor allele frequency ≥0.1% in EU and ≥1% in AFR, followed by random effects meta‐analysis (Plink v2.0; GWAMA v2.2.2). An APOE*4‐by‐SNP interaction model was also evaluated. GWAS performed multiple linear regression on an AD‐age score (Le Guen & Belloy et al. 2021; **Figure‐1B**), adjusting for APOE*4/APOE*2 dosage, the first five genetic principal components (PC‐AiR; GENESIS; R v3.6), and array/sequencing center. Variants were filtered to be covered on at least half of the individuals per stratum and on both EU as well as AFR ancestry datasets.

**Result:**

We found 1 novel genome‐wide significant locus in the APOE*4‐by‐SNP interaction GWAS (**Figure‐2**), with lead variant rs17042744 located intronic on a long non‐coding RNA (ENSG00000233005) and 558kb away from *APOB*. This variant showed disparate allele frequencies across the studied populations, yet was consistently protective in APOE*4 carriers and risk‐increasing in APOE*4 non‐carriers (**Figure‐3**).

**Conclusion:**

We show the relevance of expanding beyond non‐Hispanic white samples to identify genetic risk variants that may modulate the effect of APOE*4 on AD risk, identifying a risk locus with substantially elevated frequencies in African compared to European ancestry. The locus harbored *APOB*, a ligand for the *LDL* receptor, regulator of plasma cholesterol levels, and known to have mutations in its regulatory region that cause hypobetalipoproteinemia and hypercholesterolemia. Ongoing work is exploring replication and computational functional genomics follow‐up work.